# Current Evidence of Digital and Hand Replantation Following Cold Ischemia or Cryopreservation: A Systematic Review

**DOI:** 10.1002/micr.70323

**Published:** 2026-11-11

**Authors:** Alec J. Chen, Elizabeth K. Laning, Myiah P. Quach, Justin M. LeBlanc, Brandon P. LeBlanc, Weifeng Zeng, Aaron M. Dingle, Samuel O. Poore

**Affiliations:** ^1^ Division of Plastic and Reconstructive Surgery University of Wisconsin School of Medicine and Public Health Madison Wisconsin USA; ^2^ Krieger School of Arts and Sciences Johns Hopkins University Baltimore Maryland USA

**Keywords:** cryopreservation, finger injuries, hand injuries, replantation

## Abstract

**Background:**

Digit and hand amputations are debilitating traumatic injuries associated with severely diminished quality of life. Although replantation is possible, it is logistically challenging due to the time sensitivity of these procedures. The upper limit of cold ischemia (4°C) for digit and hand replantation is 24 and 12 h, respectively. This review aims to summarize replantation outcomes following preservation below 4°C and/or beyond the ischemic limits.

**Methods:**

The PubMed, Embase, Web of Science, and Scopus databases were searched from inception until December 2025 to identify all manuscripts reporting digit or hand replantation following preservation at ≤ 10°C or exceeding 6 h.

**Results:**

Of 139 unique citations, 10 (*n* = 255 patients, 287 replantation) were included. The mean age was 29.7 ± 8.2 years. The duration of preservation was 6 to < 24 h (*n* = 242/287, 84.3%), 24 to < 36 h (*n* = 29/287, 10.1%), 36 to < 48 h (*n* = 8/287, 2.8%), 48 to < 96 h (*n* = 4/287, 1.4%), or 5 to 81 days (*n* = 4/287, 1.4%). Preservation temperature ranges included hyperthermic (≥ 38°C; *n* = 1/287, 0.3%), sub‐normothermic (20°C to < 35°C; *n* = 84/287, 29.3%), cold ischemia (0°C to < 10°C; *n* = 197/287, 68.6%), high subzero (−20°C to < 0°C; *n* = 1/287, 0.3%), and cryopreservation (< −196°C to −80°C; *n* = 4/287, 1.4%). The overall replantation survival rate was 81.5% (*n* = 234/287). Two manuscripts reported four successful digit replantation cases following cryopreservation (−196°C) up to 81 days.

**Conclusion:**

Further investigation into modalities for extending digit and hand preservation times may improve outcomes, expand patient candidacy, and enable elective scheduling of digit and hand replantation.

## 1 Introduction

Digit and hand amputations cause a significant healthcare burden due to the significant quality of life impacts on patients. Epidemiology studies have shown that it predominately affects males (89.1%) and is most commonly caused by occupational injuries (38.7%) (Mayrhofer‐Schmid et al. 2025). The estimated success rate of digit replantation is 70%–90% (Yamano 1985; Chen et al. 1994; Kim et al. 1996; Patradul et al. 1998). Although digit replantation can restore sensation, function, and cosmetic appearance, timely intervention is crucial (Hattori et al. 2006). The currently accepted upper limit is 12 h of warm or 24 h of cold ischemia time for digits and 6 h of warm or 12 h of cold ischemia for more proximal amputations (i.e., hand amputation) (Wolfe and Wang 2015). Given the short window of time, there are substantial barriers for timely intervention. One factor is the considerable variation in replantation outcomes across different hospitals due to the limited supply of microsurgeons capable of performing these procedures (Billig et al. 2021). As such, if patients require a transfer to another hospital for care, that could significantly prolong the ischemia time.

This narrow window makes digit replantation a challenging logistical problem. The current understanding is that prolonged ischemia may lead to ischemia–reperfusion injury upon the return of oxygenated blood flow, which is characterized by cellular injury and necrosis (Widgerow 2014). One possible solution to lengthen the preservation time is to lower storage temperatures. There have been reported cases of successful digit replantation following multiple days of preservation in liquid nitrogen at −196°C (Wang et al. 2019, 2020). By lowering the preservation temperature, the generation of reactive oxygen species diminishes, thus causing reduced tissue damage (Sapega et al. 1988).

Given the evidence of digit replantation procedures involving preservation times that exceed the clinically accepted upper limits of ischemia times, this systematic review aims to summarize the current literature of cases involving cold ischemia or subzero preservation modalities with extended preservation times (> 6 h). This review aims to improve patient outcomes by synthesizing the current evidence on preservation modalities and highlighting opportunities for technological innovations to extend preservation times for digit and hand preservation.

## 2 Methods

### 2.1 Search Strategy

From inception to December 20, 2025, the PubMed, Embase, Web of Science, and Scopus repositories were searched to identify all articles describing digit or hand replantation following an ischemic time over 6 h or at a temperature of ≤ 10°C. The comprehensive search strategy is provided in Supporting Information S1.

### 2.2 Article Appraisal

The inclusion criteria consisted of studies describing patients of any age who underwent digit or hand replantation following preservation at ≤ 10°C or for a period over 6 h. Acceptable study designs included case reports, case series, case–control studies, cross‐sectional studies, cohort studies, and randomized controlled trials. Studies with only a sub‐set of patients that fit the inclusion criteria were still included if stratified data were available for the patients of interest. Only articles available in English were included.

The exclusion criteria were defined as any studies with patients who underwent digit replantation without mention of preservation modality, a preservation time of under 6 h, and preservation at a temperature greater than 10°C. Letters, editorials, correspondences, and review papers lacking original data, as well as papers not available in English, were all excluded.

All citations were uploaded to Covidence (Veritas Health Innovation, Melbourne, Australia) for screening (Covidence Systematic Review Software, n.d.). Based on the pre‐defined inclusion and exclusion criteria, two rounds of screening (title‐abstract and full‐text) was independently completed by two reviewers (J.M.L., B.P.L.). Any conflicts were resolved by a third reviewer (A.J.C.).

### 2.3 Data Extraction

Study characteristics were collected, which included the study design and whether the study was retrospective or prospective. Patient characteristics included the number of patients, age, gender, mechanism of injury, and total replantation procedures. The primary outcome was the reported success of digit or hand replantation following the ischemic preservation period. Secondary outcomes included the method of preservation, temperature of preservation, length of preservation, replantation survival, replantation failure, postoperative complications, and length of follow‐up.

Preservation temperature was categorized into the following: hyperthermic (≥ 38°C), normothermic (35°C to < 38°C), sub‐normothermic (20°C to < 35°C), moderate hypothermic (10°C to < 20°C), cold ischemia (0°C to < 10°C), high subzero (−20°C to < 0°C), deep freezing (−80°C to < −20°C), and cryopreservation (−196°C to < −80°C). Two of four possible reviewers (J.M.L., B.P.L., A.J.C., M.P.Q.) independently conducted the data extraction, with any conflicts being resolved by discussion between reviewers.

### 2.4 Risk of Bias Assessment

A risk of bias assessment was conducted for all included studies using the Joanna Briggs Institute (JBI) critical appraisal tools for case reports, case series, cohort studies, case–control studies, cross‐sectional studies, and randomized controlled trials (Munn et al. 2020). A summary of all key outcomes was also assessed. Two reviewers (J.M.L., B.P.L.) independently assessed all studies, with conflicts being resolved by a third reviewer (A.J.C).

### 2.5 Statistical Analysis

Using Microsoft Excel, outcomes of interest were analyzed using descriptive statistics across all included studies (Microsoft Corporation, n.d.). All continuous variables were reported as mean ± standard deviation (SD) and categorical variables as *n* (%). Using Prism GraphPad, a Fisher's exact test was utilized to compare categorical variables at the *α* = 0.05 level (GraphPad Software, n.d.). This systematic review was conducted following the 2020 Preferred Reporting Items for Systematic Reviews and Meta‐Analyses (PRISMA) guidelines. This study was registered with PROSPERO (CRD420261279461).

## 3 Results

### 3.1 Article Selection

The search strategy yielded a total of 296 citations across PubMed, Embase, Web of Science, and Scopus. Upon the removal of duplicates, 139 studies were assessed for title‐abstract screening, of which 97 were deemed irrelevant. The remaining 42 studies were evaluated through full‐text review. After the exclusion of 32 articles, a total of 10 manuscripts were included in this systematic review (Figure 1) (Wang et al. 2019, 2020; Li et al. 2008; Lin et al. 2010; May Jr. et al. 1986; VanderWilde et al. 1992; Watson et al. 2018; Wei et al. 1988; Woo et al. 2015; Zhou and Zhang 1991).

**FIGURE 1:**
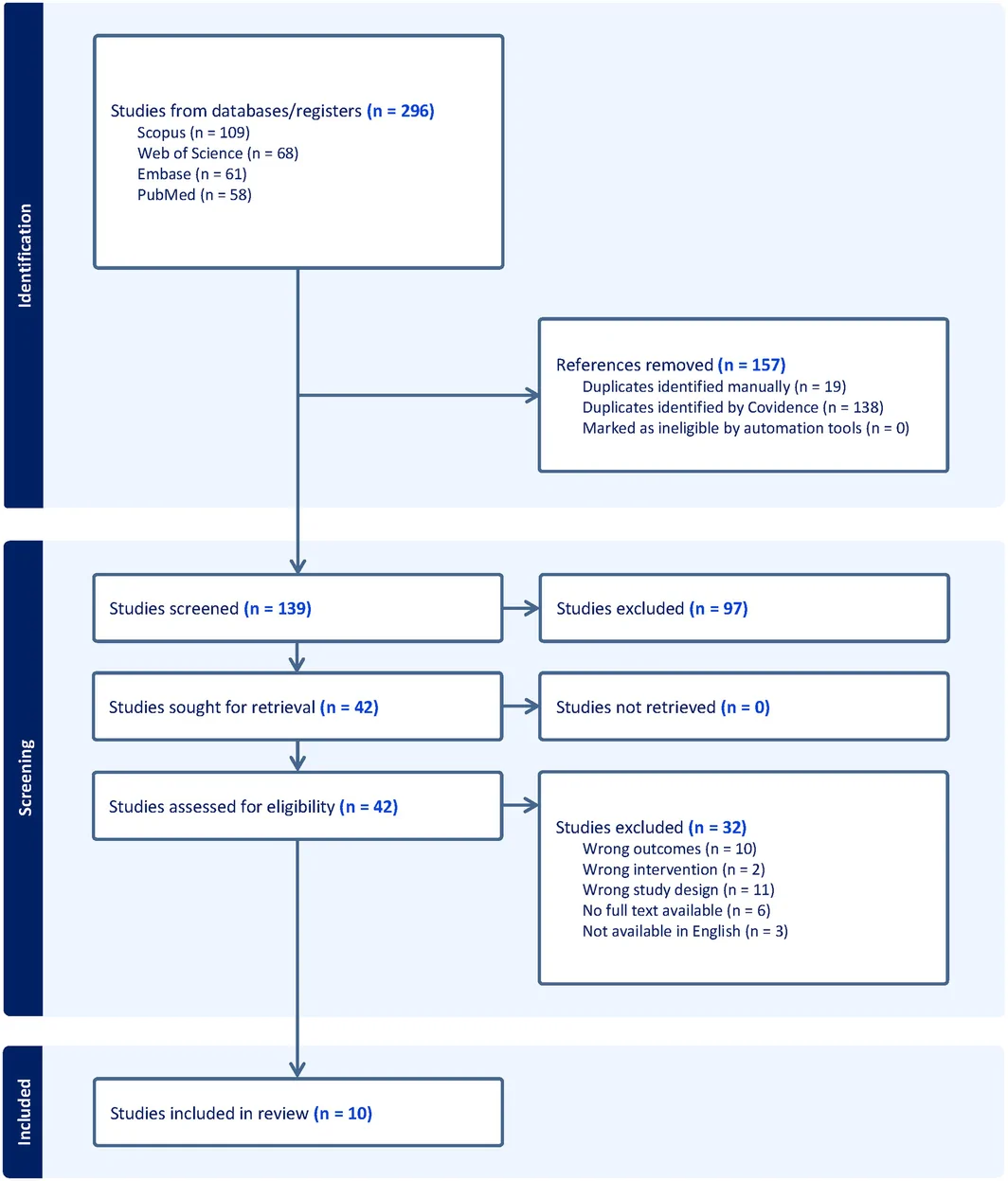
PRISMA diagram of article selection.

### 3.2 Summary of Study Findings

A total of 255 patients were included (Table 1). The mean age was 29.7 ± 8.2 years. There were 155 (60.8%) males and 100 (39.2%) females. Mechanisms of injuries included crush injury or avulsion (*n* = 105/255, 41.2%), sharp amputation (*n* = 80/255, 31.4%), blunt amputation (*n* = 55/255, 21.6%), guillotine or crush injury (*n* = 14/255, 5.5%), and human bite (*n* = 1/255, 0.4%).

**TABLE 1:** Summary of study findings.

	Overall cohort	Digit replantation	Hand replantation
Study information			
Total studies	10	8	4
Studies reporting digit replantation only	6/10 (60%)	—	—
Studies reporting hand replantation only	2/10 (20%)	—	—
Studies reporting both digit and hand replantation	2/10 (20%)	—	—
Case series	6/10 (60%)	6/8 (75%)	2/4 (50%)
Case reports	4/10 (4%)	2/8 (25%)	2/4 (50%)
Retrospective studies	10/10 (100%)	8/8 (100%)	4/4 (100%)
Total patients and procedures			
Total patients	255	250/255 (98.0%)	7/255 (2.8%)
Total patients with digit replantation only	248	—	—
Total patients with hand replantation only	5	—	—
Total patients with both digit and hand replantation	2	—	—
Total replantation procedures	287	279/287 (97.2%)	8/287 (2.8%)
Patient demographics			
Mean age (years)	29.7 ± 8.2	28.2 ± 6.5	34.6 ± 21.0
Age range	1–67	1–67	15–54
Male	155/255 (60.8%)	151/250 (60.4%)	6/7 (85.7%)
Female	100/255 (39.2%)	99/250 (39.6%)	1/7 (14.3%)
Mechanisms of injury			
Crush or avulsion	105/255 (41.2%)	105/250 (42%)	0/7 (0%)
Sharp amputation	80/255 (31.4%)	77/250 (30.8%)	5/7 (71.4%)
Blunt amputation	55/255 (21.6%)	55/250 (22%)	0/7 (0%)
Guillotine or crush	14/255 (5.5%)	12/250 (4.8%)	2/7 (28.6%)
Human bite	1/255 (0.4%)	1/250 (0.4%)	0/7 (0%)
Preservation temperature			
Hyperthermic (≥ 38°C)	1/287 (0.3%)	1/279 (0.4%)	0/8 (0%)
Normothermic (35°C to < 38°C)	0/287 (0%)	0/279 (0%)	0/8 (0%)
Sub‐normothermic (20°C to < 35°C)	84/287 (29.3%)	84/279 (30.1%)	0/8 (0%)
Moderate hypothermic (10°C to < 20°C)	0/287 (0%)	0/279 (0%)	0/8 (0%)
Cold ischemia (0°C to < 10°C)	197/287 (68.6%)	189/279 (67.7%)	8/8 (100%)
High subzero (−20°C to < 0°C)	1/287 (0.3%)	1/279 (0.4%)	0/8 (0%)
Deep freezing (−80°C to < −20°C)	0/287 (0%)	0/279 (0%)	0/8 (0%)
Cryopreservation (−196°C to < −80°C)	4/287 (1.4%)	4/279 (1.4%)	0/8 (0%)
Preservation duration			
6 to < 24 h	242/287 (84.3%)	238/279 (85.3%)	4/8 (50%)
24 to < 36 h	29/287 (10.1%)	26/279 (9.3%)	3/8 (37.5%)
36 to < 48 h	8/287 (2.8%)	8/279 (2.9%)	0/8 (0%)
48 to < 96 h (2 to < 4 days)	4/287 (1.4%)	3/279 (1.1%)	1/8 (12.5%)
5 to 81 days	4/287 (1.4%)	4/279 (1.4%)	0/8 (0%)
Replantation outcomes			
Replantation survival	234/287 (81.5%)	227/279 (81.4%)	7/8 (87.5%)
Replantation failure	53/287 (18.5%)	52/279 (18.6%)	1/8 (12.5%)
Overall complications of successful replantation	8/287 (2.8%)	8/279 (2.9%)	0/8 (0%)
Overall complications	61/287 (21.3%)	60/279 (21.5%)	1/8 (12.5%)
Causes of replantation failure			
Tissue necrosis	39/287 (13.6%)	39/279 (14.0%)	0/8 (0%)
Tissue ischemia	1/287 (0.3%)	1/279 (0.4%)	0/8 (0%)
Venous congestion	1/287 (0.3%)	0/279 (0%)	1/8 (12.5%)
NR	12/287 (4.2%)	12/279 (4.3%)	0/8 (0%)
Overall complications of successful replantation			
Thrombosis	5/287 (1.7%)	5/279 (1.8%)	0/8 (0%)
Edema	3/287 (1.0%)	3/279 (1.1%)	0/8 (0%)
Follow‐up details			
Mean follow‐up (years)	0.6 ± 1.5	0.4 ± 1.5	2.3 ± 3.3
Follow‐up range	4 weeks–15 years	4 weeks–15 years	24–30 months

*Note:* Patient characteristics and outcomes were divided with a denominator of either 255 (total patients) or 287 (total procedures).

Abbreviation: NR, not reported.

A total of 287 replantation procedures were performed, consisting of digit (*n* = 279/287, 97.2%) or hand replantation (*n* = 8/287, 2.8%). The ranges of preservation temperatures included hyperthermic (*n* = 1/287, 0.3%), sub‐normothermic (*n* = 84/287, 29.3%), cold ischemia (*n* = 197/287, 68.6%), high subzero (*n* = 1/287, 0.3%), and cryopreservation (*n* = 4/287, 1.4%). No cases involved preservation in the normothermic, moderate hypothermic, or deep‐freezing temperature ranges. The ischemic duration of preservation ranged from 6 to < 24 h (*n* = 242/287, 84.3%), 24 to < 36 h (*n* = 29/287, 10.1%), 36 to < 48 h (*n* = 8/287, 2.8%), 48 to < 96 h (*n* = 4/287, 1.4%), or 5 to 81 days (*n* = 4/287, 1.4%). The details regarding each study's specific preservation details can be found in Table 2.

**TABLE 2:** Outcomes of replantation by individual study.

First author, year	Method of preservation	Survival rate of replantation
Li et al. (2008)	Dry storage at 2°C–6°C for > 8 h, 8–16 h, or > 16 h (*n* = 106 patients, 106 digit replants)	Digit: 93/106 (87.7%)
	Dry storage at room temperature for > 8 h, 8–16 h, or > 16 h (*n* = 84 patients, digit 84 replants)	Digit: 67/84 (79.8%)
	Preservation in saline or ethanol at 2°C–6°C for > 8 h, 8–16 h, or > 16 h (*n* = 21 patients, 21 digit replants)	Digit: 12/21 (57.1%)
Lin et al. (2010)	Warm and cold ischemia for > 24 h (*n* = 14 patients, 23 digit replants, 2 hand replants)	Digit: 15/23 (65.2%); hand: 1/2 (50%)
May Jr. et al. (1986)	Cold ischemia via iced saline sponges for up to 40 h (*n* = 1 patient, 8 digit replants)	Digit: 7/8 (87.5%)
VanderWilde et al. (1992)	Refrigeration at 2°C–5°C with intermittent exposure up to 9°C for 54 h (*n* = 1 patient, 1 hand replant)	Hand: 1/1 (100%)
Wang et al. (2019)	Initial cold storage at 4°C for 15 h, followed by controlled‐rate freezing at −80°C for 24 h, and liquid nitrogen cryopreservation at −196°C for 5 days (7 total days; *n* = 1 patient, 1 digit replant) or 79 days (81 total days; *n* = 1 patient, 1 digit replant)	Digit: 2/2 (100%)
Wang et al. (2020)	Incubation at 4°C for 2 h, followed by 1°C/min controlled freezing to −80°C, then liquid nitrogen cryopreservation (−196°C) for 10 days (*n* = 1 patient, 1 digit replant) or 30 days (*n* = 1 patient, 1 digit replant)	Digit: 2/2 (100%)
Watson et al. (2018)	Plastic bag in cool‐box with ice slurry at < 4°C for 18 h (*n* = 1 patient, 1 hand replant)	Hand: 1/1 (100%)
Wei et al. (1988)	Wrapped in saline‐soaked gauze, preserved in ice bag (< 4°C) for 84 h, 86 h, or 94 h (*n* = 1 patient, 3 digit replants)	Digit: 3/3 (100%)
Woo et al. (2015)	Warm ischemia for an average of 2 h (1.5–5) and cold ischemia at 4°C for an average of 7.5 h (5–9.5 h) (*n* = 20 patients, 28 digit replants, 4 hand replants)	Digit: 24/28 (85.7%); hand: 4/4 (100%)
Zhou and Zhang (1991)	Left at the site of injury at −20°C for 1.5 h then refrigerated at 4°C for 15.5 h (*n* = 1 patient, 1 digit replant)	Digit: 1/1 (100%)
	Left at site of injury at 57°C for 4 h then refrigerated at 4°C for 14 h (*n* = 1 patient, 1 digit replant)	Digit: 1/1 (100%)

The overall replantation survival rate was 81.5% (*n* = 234/287). Replantation failure (*n* = 53/287, 18.5%) was due to tissue necrosis (*n* = 39/287, 13.6%), tissue ischemia (*n* = 1/287, 0.3%), venous congestion (*n* = 1/287, 0.3%), or an unspecified reason (*n* = 12/287, 4.2%). The survival rate of digit replantation stratified by preservation temperature and duration can be found in Table 3. The mean follow‐up was 0.6 ± 1.5 years. Study outcomes by individual article can be found in Supporting Information S2.

**TABLE 3:** Digit replantation survival by preservation temperature and duration.

	6 to < 24 h	24 to < 36 h	36 to < 48 h	48 to < 96 h (2 to < 4 days)	5 to 100 days	Overall survival
Hyperthermic (≥ 38°C)	1/1 (100%)					1/1 (100%)
Normothermic (35°C to < 38°C)						
Sub‐normothermic (20°C to < 35°C)	67/84 (79.8%)					67/84 (79.8%)
Moderate hypothermic (10°C to < 20°C)						
Cold ischemia (0°C to < 10°C)	126/152 (82.9%)	18/26 (69.2%)	7/8 (87.5%)	3/3 (100%)		154/189 (81.5%)
High subzero (−20°C to < 0°C)	1/1 (100%)					1/1 (100%)
Deep freezing (−80°C to <‐20°C)						
Cryopreservation (−196°C to <‐80°C)					4/4 (100%)	4/4 (100%)
Overall survival	195/238 (82.3%)	18/26 (69.2%)	7/8 (87.5%)	3/3 (100%)	4/4 (100%)	227/279 (81.4%)

### 3.3 Preservation Modalities Exceeding 24 h or Involving Storage Below 0°C

A total of six studies involved an ischemic preservation duration which exceeded 24 h (Lin et al. 2010; May Jr. et al. 1986; VanderWilde et al. 1992; Wang et al. 2019, 2020; Wei et al. 1988; Wang et al. 2019, 2020; Lin et al. 2010; May Jr. et al. 1986; VanderWilde et al. 1992; Wei et al. 1988). Lin et al. (2010) described a combination of warm and cold ischemia exceeding 24 h, with 16 of 25 replantations surviving (Lin et al. 2010). May Jr. et al. (1986) details 7 of 8 replantations surviving following cold ischemia up to 40 h in one patient (May Jr. et al. 1986). VanderWilde et al. (1992) involved a case report of a successful replantation in one patient after 54 h in cold ischemia (VanderWilde et al. 1992). Wang et al. (2019) and Wang et al. (2020) both reported successful replantation following controlled freezing and extended cryopreservation at −196°C, with delayed replantation ranging from 5 to 81 days after the amputation injury (Wang et al. 2019, 2020). Wei et al. (1988) was a case report of three replants performed at 84, 86, and 94 h post‐amputation, during which digits were stored at 4°C (Wei et al. 1988).

A total of five cases across three studies reported evidence of preservation below 0°C (Wang et al. 2019, 2020; Zhou and Zhang 1991) (Wang et al. 2019, 2020). Zhou and Zhang (1991) reported a case of digit replantation survival following 1.5 h of exposure to −20°C with subsequent storage at 4°C for 15.5 h (Zhou and Zhang 1991).

Compared to the digit replantation survival rate (*n* = 126/152, 82.9%) of cold ischemia storage for 6 to < 24 h (reference group), hyperthermic storage for 6 to < 24 h (*n* = 1/1, 100%, *p* = 1), sub‐normothermic storage for 6 to < 24 h (*n* = 67/84, 79.8%, *p* = 0.6), cold ischemia storage for 24 to < 36 h (*n* = 18/26, 69.2%, *p* = 0.1), cold ischemia storage for 36 to < 48 h (*n* = 7/8, 87.5%, *p* = 1), cold ischemia storage for 48 to < 96 h (*n* = 3/3, 100%, *p* = 1), high subzero storage for 6 to < 24 h (*n* = 1/1, 100%, *p* = 1), and cryopreservation for 5 to 100 days (*n* = 4/4, 100%, *p* = 1) showed no statistical difference (Table 4).

**TABLE 4:** Comparison of digit preservation modalities to cold ischemia for 6–< 24 h.

Preservation temperature, duration	Survival	*p*
Cold ischemia (0°C to < 10°C), 6 to < 24 h	126/152 (82.9%)	Reference
Hyperthermic (≥ 38°C), 6 to < 24 h	1/1 (100%)	*p* > 0.9999
Sub‐normothermic (20°C to < 35°C), 6 to < 24 h	67/84 (79.8%)	*p* = 0.5987
Cold ischemia (0°C to < 10°C), 24 to < 36 h	18/26 (69.2%)	*p* = 0.1106
Cold ischemia (0°C to < 10°C), 36 to < 48 h	7/8 (87.5%)	*p* > 0.9999
Cold ischemia (0°C to < 10°C), 48 to < 96 h	3/3 (100%)	*p* > 0.9999
High subzero (−20°C to < 0°C), 6 to < 24 h	1/1 (100%)	*p* > 0.9999
Cryopreservation (−196°C to <‐80°C), 5 to 100 days	4/4 (100%)	*p* > 0.9999

### 3.4 Risk of Bias Assessment

Six articles were deemed to have a “high” overall certainty with clear descriptions of the inclusion criteria, outcome measurements, consecutive and complete inclusion, and appropriate follow‐up (Supporting Information S3). Four articles were rated with a “moderate” overall certainty due to unclear reporting of complete and consecutive inclusion. For case series, appropriate statistical analyses were considered to not be applicable due to the descriptive reporting of patient data. All outcomes were rated with a “high” overall certainty except for causes of replantation failure, which was rated with a “moderate” overall certainty due to two studies not reporting the reason for replantation failure.

## 4 Discussion

This systematic review of 10 articles found evidence of successful digit replantation with preservation times that exceeded 24 h. Furthermore, there were four reported cases of digits stored in cryopreservation at −196°C with delayed replantation of up to 81 days. These interventions may serve as the framework for the development of standardized protocols for extended preservation of hands and digits for delayed replantation.

Across all cases, the overall survival rate was 81.5%, with a sub‐group of digit replantation in cold ischemia for 24–36 h reporting a 69.2% survival rate. The overall survival rate aligns with the reported range of successful digit replantation (70%–90%) in the literature (Yamano 1985; Chen et al. 1994; Kim et al. 1996; Patradul et al. 1998). However, this may reflect publication bias towards successful replantation and thus, it may not capture many instances of failed replantation. The favorable outcomes in these cases may reflect the prognosis of the injury types because the decision to undergo digit or hand replantation is highly dependent on the severity and mechanism of injury. For instance, severely crushed or mangled injuries may not be feasible for replantation (Lin et al. 2010). In contrast, many of these cases included in this review were due to sharp or blunt amputations.

Furthermore, some studies recommend that replantation should not be pursued in patients presenting with life‐threatening injuries, arteriosclerotic vessels, or poor mental health (Lin et al. 2010). This may be an area better suited for delayed replantation as patients can be stabilized before they are ready for elective digit replantation, supported by cryopreservation enabling the storage of the digit up to 81 days until the patient was ready to undergo replantation (Wang et al. 2019). Another benefit of delayed replantation technology may be patients in regions with limited access to microsurgeons. A primary hospital may be able to freeze their digit while transferring care to highly specialized providers. Transforming digit replantation into more elective and scheduled procedures, as opposed to emergent procedures, may also reduce the burden on healthcare providers. A survey of hand surgeons from 2022 reported a satisfaction rate of 46% for those taking hand call (Douleh et al. 2022).

The limited sample size of just four cases of cryopreservation of digits warrants further investigation into the feasibility of this protocol for standardized clinical practice. This is due to the possible increased risk of post‐operative complications following extended cryopreservation. Two of the biggest challenges faced by preservation below 0°C in a similar field of organ transplantation are the damaging effects of ice crystal formation and rewarming injuries (Tolouee et al. 2022; William and Acker 2021). Intracellular ice formation may cause mechanical damage by piercing outer organ membranes or tearing apart cells (Pegg 2015). Furthermore, as extracellular ice forms, the decreased osmotic pressure draws water from the intracellular to the extracellular compartment, causing cellular shrinkage and dehydration (Muldrew and McGann 1994). Another challenge of organ cryopreservation is achieving uniform cooling and rewarming, due to thermal gradients across the large volume of an organ (Sun et al. 2026; Solanki et al. 2017). Although a slow rewarming process enables even temperature distribution, this may increase the risk of recrystallization injuries around freezing temperature (Chang and Zhao 2021). This is due to smaller ice crystals melting and recrystallizing on existing ice crystals, thus forming larger ice crystals and possibly damaging the organ (Chang and Zhao 2021).

In digit preservation, the mechanisms of injury are unclear, but may be less damaging due to the anatomical differences from organs. First, one possible explanation may be that the smaller size of the digit may reduce associated damage from uneven rewarming as smaller volume will have a reduced temperature gradient difference. Furthermore, another possible explanation may be that digits have reduced vasculature and less complicated physiological function than organs (i.e., renal function in kidneys). This may allow for a higher threshold for tissue damage and injuries to be more reversible. With limited cases of digit replantation following cryopreservation, further research is needed to determine the physiological changes during the storage process.

There are several limitations to this study. First, the heterogeneity of preservation protocols makes it difficult to compare the effectiveness of specific preservation modalities. Second, variation in patient follow‐up duration may have influenced the reported replantation survival rate. Third, publication bias may have resulted in an overrepresentation of successful replantation, thereby introducing survivorship bias. Fourth, a single study contributed 211 of 255 included patients, which may contribute to a bias arising from a single study. Fifth, all included studies were case reports or case series with comparison groups, limiting the quality of evidence. Lastly, patients of all ages were included, which may have affected clinical outcomes due to younger patients generally exhibiting greater healing and regenerative capabilities.

## 5 Conclusion

This review found evidence of successful replantation with preserved digits exceeding 24 h of cold ischemia, as well as up to 81 days under cryopreservation at −196°C. Additional research is needed to investigate prolonged preservation times, which may improve patient outcomes by reducing ischemic injury, expand patient candidacy by allowing delayed replantation, and reduce the healthcare burden by enabling elective scheduling of replantation.

## Author Contributions

A.J.C., E.K.L., W.Z., A.M.D., and S.O.P. designed the study. A.J.C., J.M.L., and B.P.L. conducted screening. A.J.C., M.P.Q., J.M.L., and B.P.L. collected the data. A.J.C., E.K.L., and M.P.Q. performed data analysis. A.J.C. and E.K.L. drafted the manuscript. All authors reviewed and approved the final manuscript.

## Funding

The authors have nothing to report.

## Disclosure

This study was presented in abstract form as an oral presentation at the Annual 2026 University of Wisconsin Plastic and Reconstructive Surgery Resident Research Day on June 17, 2026.

## Ethics Statement

The authors have nothing to report.

## Conflicts of Interest

The authors declare no conflicts of interest.

## Supporting information


**Supporting Information: S1.** Search strategy.

**Supporting Information: S2.** Outcomes of each individual study.

**Supporting Information: S3.** Risk of bias assessment.

## Data Availability

The data in this study are available in the Supporting Information.

## References

[micr70323-bib-0001] Billig, J. I. , J. S. Nasser , H. E. Cho , C. H. Chou , and K. C. Chung . 2021. “Association of Interfacility Transfer and Patient and Hospital Characteristics With Thumb Replantation After Traumatic Amputation.” JAMA Network Open 4: e2036297. 10.1001/jamanetworkopen.2020.36297. PMC7859845 33533928

[micr70323-bib-0002] Chang, T. , and G. Zhao . 2021. “Ice Inhibition for Cryopreservation: Materials, Strategies, and Challenges.” Advanced Science (Weinheim) 8: 2002425. 10.1002/advs.202002425. PMC7967093 33747720

[micr70323-bib-0003] Chen, C. T. , F. C. Wei , H. C. Chen , et al. 1994. “Distal Phalanx Replantation.” Microsurgery 15: 77–82. 10.1002/micr.1920150118. 8133774

[micr70323-bib-0004] Covidence Systematic Review Software . n.d. “Veritas Health Innovation.” Melbourne. Australia. www.covidence.org.

[micr70323-bib-0005] Douleh, D. G. , K. Ipaktchi , and A. Lauder . 2022. “Hand Call Practices and Satisfaction: Survey Results From Hand Surgeons in the United States.” Journal of Hand Surgery 47: e1121–e1129. 10.1016/j.jhsa.2021.08.021. 34756619

[micr70323-bib-0006] GraphPad Software . n.d. “GraphPad Prism Version 11.1.0 for Mac OS X.” Boston. Massachusetts USA. www.graphpad.com.

[micr70323-bib-0007] Hattori, Y. , K. Doi , K. Ikeda , and E. P. Estrella . 2006. “A Retrospective Study of Functional Outcomes After Successful Replantation Versus Amputation Closure for Single Fingertip Amputations.” Journal of Hand Surgery 31: 811–818. 10.1016/j.jhsa.2006.02.020. 16713848

[micr70323-bib-0008] Kim, W. K. , J. H. Lim , and S. K. Han . 1996. “Fingertip Replantations: Clinical Evaluation of 135 Digits.” Plastic and Reconstructive Surgery 98: 470–476. 10.1097/00006534-199609000-00017. 8700984

[micr70323-bib-0009] Li, J. , Z. Guo , Q. Zhu , et al. 2008. “Fingertip Replantation: Determinants of Survival.” Plastic and Reconstructive Surgery 122: 833–839. 10.1097/PRS.0b013e318180ed61. 18766047

[micr70323-bib-0010] Lin, C. H. , N. Aydyn , Y. T. Lin , et al. 2010. “Hand and Finger Replantation After Protracted Ischemia (More Than 24 Hours).” Annals of Plastic Surgery 64: 286–290. 10.1097/SAP.0b013e3181b0bb37. 20179474

[micr70323-bib-0011] May, J. W., Jr. , C. A. Hergrueter , and R. H. Hansen . 1986. “Seven‐Digit Replantation: Digit Survival After 39 Hours of Cold Ischemia.” Plastic and Reconstructive Surgery 78: 522–525. 10.1097/00006534-198610000-00017. 3763731

[micr70323-bib-0012] Mayrhofer‐Schmid, M. , M. Aman , A. Stolle , et al. 2025. “Analysis of Epidemiology, Etiology and Injury Patterns in 2,179 Digit Amputations.” Scientific Reports 15: 32657. 10.1038/s41598-025-18983-y. PMC12457628 40987779

[micr70323-bib-0013] Microsoft Corporation . n.d. “Microsoft Excel.” https://office.microsoft.com/excel.

[micr70323-bib-0014] Muldrew, K. , and L. E. McGann . 1994. “The Osmotic Rupture Hypothesis of Intracellular Freezing Injury.” Biophysical Journal 66: 532–541. 10.1016/s0006-3495(94)80806-9. PMC1275720 8161706

[micr70323-bib-0015] Munn, Z. , T. H. Barker , S. Moola , et al. 2020. “Methodological Quality of Case Series Studies: An Introduction to the JBI Critical Appraisal Tool.” JBI Evidence Synthesis 18: 2127–2133. 10.11124/JBISRIR-D-19-00099. 33038125

[micr70323-bib-0016] Patradul, A. , C. Ngarmukos , and V. Parkpian . 1998. “Distal Digital Replantations and Revascularizations. 237 Digits in 192 Patients.” Journal of Hand Surgery 23: 578–582. 10.1016/s0266-7681(98)80005-3. 9821597

[micr70323-bib-0017] Pegg, D. E. 2015. “Principles of Cryopreservation.” Methods in Molecular Biology 1257: 3–19. 10.1007/978-1-4939-2193-5_1. 25428001

[micr70323-bib-0018] Sapega, A. A. , R. B. Heppenstall , D. P. Sokolow , et al. 1988. “The Bioenergetics of Preservation of Limbs Before Replantation. The Rationale for Intermediate Hypothermia.” Journal of Bone and Joint Surgery. American Volume 70: 1500–1513. 3198676

[micr70323-bib-0019] Solanki, P. K. , J. C. Bischof , and Y. Rabin . 2017. “Thermo‐Mechanical Stress Analysis of Cryopreservation in Cryobags and the Potential Benefit of Nanowarming.” Cryobiology 76: 129–139. 10.1016/j.cryobiol.2017.02.001. PMC5651684 28192076

[micr70323-bib-0020] Sun, W. , X. Dong , X. Wang , P. Yuan , and M. Gong . 2026. “Numerical Thermal Analysis and Optimization of Vitrification Cooling Strategies for Porcine Heart Cryopreservation.” Cryobiology 122: 105570. 10.1016/j.cryobiol.2025.105570. 41385888

[micr70323-bib-0021] Tolouee, M. , K. D. W. Hendriks , F. F. Lie , et al. 2022. “Cooling of Cells and Organs Confers Extensive DNA Strand Breaks Through Oxidative Stress and ATP Depletion.” Cell Transplantation 31: 9636897221108705. 10.1177/09636897221108705. PMC9272479 35808831

[micr70323-bib-0022] VanderWilde, R. S. , M. B. Wood , and Z. G. Zu . 1992. “Hand Replantation After 54 Hours of Cold Ischemia: A Case Report.” Journal of Hand Surgery 17: 217–220. 10.1016/0363-5023(92)90394-5. 1564265

[micr70323-bib-0023] Wang, J. , J. Lin , Y. Pei , Q. Xu , and L. Zhu . 2020. “Cryopreservation and Transplantation of Amputated Finger.” Cryobiology 92: 235–240. 10.1016/j.cryobiol.2020.01.017. 32001216

[micr70323-bib-0024] Wang, Z. , L. Zhu , W. Kou , et al. 2019. “Replantation of Cryopreserved Fingers: An ‘Organ Banking’ Breakthrough.” Plastic and Reconstructive Surgery 144: 679–683. 10.1097/PRS.0000000000005979. 31461026

[micr70323-bib-0025] Watson, L. , H. Beem , K. Rowe , et al. 2018. “Late Hand Replantation With Restoration of Intrinsic Muscle Function: A Case and Review of the Literature.” European Journal of Plastic Surgery 41: 725–730. 10.1007/s00238-018-1416-1.

[micr70323-bib-0026] Wei, F. C. , Y. L. Chang , H. C. Chen , and C. C. Chuang . 1988. “Three Successful Digital Replantations in a Patient After 84, 86, and 94 Hours of Cold Ischemia Time.” Plastic and Reconstructive Surgery 82: 346–350. 10.1097/00006534-198808000-00026. 3399566

[micr70323-bib-0027] Widgerow, A. D. 2014. “Ischemia‐Reperfusion Injury: Influencing the Microcirculatory and Cellular Environment.” Annals of Plastic Surgery 72: 253–260. 10.1097/SAP.0b013e31825c089c. 23241775

[micr70323-bib-0028] William, N. , and J. P. Acker . 2021. “High Sub‐Zero Organ Preservation: A Paradigm of Nature‐Inspired Strategies.” Cryobiology 102: 15–26. 10.1016/j.cryobiol.2021.04.002. 33905707

[micr70323-bib-0029] Wolfe, V. M. , and A. A. Wang . 2015. “Replantation of the Upper Extremity: Current Concepts.” Journal of the American Academy of Orthopaedic Surgeons 23: 373–381. 10.5435/JAAOS-D-14-00039. 26001429

[micr70323-bib-0030] Woo, S. H. , H. J. Cheon , Y. W. Kim , D. H. Kang , and H. J. Nam . 2015. “Delayed and Suspended Replantation for Complete Amputation of Digits and Hands.” Journal of Hand Surgery 40: 883–889. 10.1016/j.jhsa.2015.01.006. 25746146

[micr70323-bib-0031] Yamano, Y. 1985. “Replantation of the Amputated Distal Part of the Fingers.” Journal of Hand Surgery 10: 211–218. 10.1016/s0363-5023(85)80107-6. 3980933

[micr70323-bib-0032] Zhou, G. H. , and W. D. Zhang . 1991. “Replantation of Severed Fingers: Two Unique Cases.” Microsurgery 12: 235–236. 10.1002/micr.1920120402. 1895931

