# Supplementary material for: Current Evidence of Digital and Hand Replantation Following Cold Ischemia or Cryopreservation: A Systematic Review
Source: Microsurgery. 2026 Nov 11;46(8):e70323. doi: 10.1002/micr.70323 (PMC13754630; doi:10.1002/micr.70323)
Supplement: Supplementary file 1 [file MICR-46-e70323-s001.docx]

**Supplementary Material 3. Risk of Bias Assessment**

**JBI Risk of Bias Assessment of Case Series**

| First Author, Year | D1 | D2 | D3 | D4 | D5 | D6 | D7 | D8 | D9 | D10 | Overall Certainty |
| --- | --- | --- | --- | --- | --- | --- | --- | --- | --- | --- | --- |
| Li 2008 | Yes | Yes | Yes | Yes | Yes | Yes | Yes | Yes | Yes | Yes | High |
| Lin 2010 | Yes | Yes | Yes | Unclear | Yes | Yes | Yes | Yes | Yes | Yes | Moderate |
| Wang 2019 | Yes | Yes | Yes | Unclear | Unclear | Yes | Yes | Yes | Yes | N/A | Moderate |
| Wang 2020 | Yes | Yes | Yes | Yes | Yes | Yes | Yes | Yes | Yes | N/A | High |
| Woo 2015 | Yes | Yes | Yes | Yes | Unclear | Yes | Yes | Yes | Yes | N/A | Moderate |
| Zhou 1991 | Yes | Yes | Yes | Unclear | Unclear | Yes | Yes | Yes | Yes | N/A | Moderate |

**D1. Were there clear criteria for inclusion in the case series?**

**D2. Was the condition measured in a standard, reliable way for all participants included in the case series?**

**D3. Were valid methods used for identification of the condition for all participants included in the case series?**

**D4. Did the case series have consecutive inclusion of participants?**

**D5. Did the case series have complete inclusion of participants?**

**D6. Was there clear reporting of the demographics of the participants in the study?**

**D7. Was there clear reporting of clinical information of the participants?**

**D8. Were the outcomes or follow-up results of cases clearly reported?**

**D9. Was there clear reporting of the presenting sites'/clinics' demographic information?**

**D10. Was statistical analysis appropriate?**

**JBI Risk of Bias Assessment of Case Reports**

| First Author, Year | D1 | D2 | D3 | D4 | D5 | D6 | D7 | D8 | Overall Certainty |
| --- | --- | --- | --- | --- | --- | --- | --- | --- | --- |
| May 1986 | Yes | Yes | Yes | Yes | Yes | Yes | Yes | Yes | High |
| VanderWilde 1992 | Yes | Yes | Yes | Yes | Yes | Yes | Yes | Yes | High |
| Watson 2018 | Yes | Yes | Yes | Yes | Yes | Yes | Yes | Yes | High |
| Wei 1988 | Yes | Yes | Yes | Yes | Yes | Yes | Yes | Yes | High |

**D1. Were patient's demographic characteristics clearly described?**

**D2. Was the patient’s history clearly described and presented as a timeline?**

**D3. Was the current clinical condition of the patient on presentation clearly described?**

**D4. Were diagnostic tests or assessment methods and the results clearly described?**

**D5. Was the intervention(s) or treatment procedure(s) clearly described?**

**D6. Was the post-intervention clinical condition clearly described?**

**D7. Were adverse events (harms) or unanticipated events identified and described?**

**D8. Does the case report provide takeaway lessons?**

**Overall Certainty of Key Outcomes**

| **Clear Reporting of Outcomes?** | | | | | | | | | | | **Overall Certainty** |
| --- | --- | --- | --- | --- | --- | --- | --- | --- | --- | --- | --- |
|  | **Li 2008** | **Lin 2010** | **May 1986** | **VanderWilde 1992** | **Wang 2019** | **Wang 2020** | **Watson 2018** | **Wei 1988** | **Woo 2015** | **Zhou 1991** |  |
| **1** | Yes | Yes | Yes | Yes | Yes | Yes | Yes | Yes | Yes | Yes | **High** |
| **2** | Yes | Yes | Yes | Yes | Yes | Yes | Yes | Yes | Yes | Yes | **High** |
| **3** | Yes | Yes | Yes | Yes | Yes | Yes | Yes | Yes | Yes | Yes | **High** |
| **4** | Yes | Yes | Yes | Yes | Yes | Yes | Yes | Yes | Yes | Yes | **High** |
| **5** | Yes | Yes | Yes | Yes | Yes | Yes | Yes | Yes | Yes | Yes | **High** |
| **6** | Yes | Yes | Yes | Yes | Yes | Yes | Yes | Yes | Yes | Yes | **High** |
| **7** | Yes | No | Yes | Yes | Yes | Yes | Yes | Yes | No | Yes | **Moderate** |
| **8** | Yes | Yes | Yes | Yes | Yes | Yes | Yes | Yes | Yes | Yes | **High** |
| **9** | Yes | Yes | Yes | Yes | Yes | Yes | Yes | Yes | Yes | Yes | **High** |
| **10** | Yes | Yes | Yes | Yes | Yes | Yes | Yes | Yes | Yes | Yes | **High** |

**1:** Age. **2:** Gender. **3:** Mechanism of Injury. **4:** Preservation Temperature. **5:** Preservation Duration. **6:** Replantation Outcomes. **7:** Causes of Replantation Failure. **8:** Overall Complications of Successful Replantation. **9:** Follow-up Details.
