# Supplementary material for: Current Evidence of Digital and Hand Replantation Following Cold Ischemia or Cryopreservation: A Systematic Review
Source: Microsurgery. 2026 Nov 11;46(8):e70323. doi: 10.1002/micr.70323 (PMC13754630; doi:10.1002/micr.70323)
Supplement: Supplementary file 2 [file MICR-46-e70323-s002.docx]

**Supplementary Material 1. Search Strategy**

**Concept 1: Digit Replantation Terms**

**Keywords and Synonyms:**

Digit replantation, finger replantation, thumb replantation

Digit reattachment, finger reattachment, thumb reattachment

Digital replantation

Finger amputation (with replantation context)

Thumb amputation (with replantation context)

Hand replantation

**Concept 2: Cryopreservation Terms**

**Keywords and Synonyms:**

Cryopreservation, cryopreserve

Freezing, frozen storage

Cold storage, cold preservation

Hypothermic preservation, hypothermia

Ice preservation

Refrigeration

Low temperature storage

Cold ischemia

Ischemia time

Ex vivo preservation

Tissue preservation

**PubMed Search Strategy**

**Set 1: Digit Replantation**

(“Replantation”[MeSH] AND (“Fingers”[MeSH] OR “Thumb”[MeSH])) OR “digit* replant*”[tw] OR “finger* replant*”[tw] OR “thumb* replant*”[tw] OR “digit replantation”[tiab:~2] OR “finger replantation”[tiab:~2] OR “thumb replantation”[tiab:~2] OR “digit* reattach*”[tw] OR “finger* reattach*”[tw] OR “thumb* reattach*”[tw] OR “digit reattachment”[tiab:~2] OR “finger reattachment”[tiab:~2] OR “thumb reattachment”[tiab:~2] OR “digital replant*”[tw] OR “digital reattach*”[tw] OR “hand replant*”[tw] OR “hand reattach*”[tw] OR (“digit amputat*”[tw] AND “replant*”[tw])

**Set 2: Cryopreservation**

(“Cryopreservation”[MeSH] OR “Hypothermia, Induced”[MeSH] OR “cryopreserv*”[tw] OR “cryo preserv*”[tw] OR “cold storage”[tiab:~2] OR “cold preservation”[tiab:~2] OR

“frozen storage”[tiab:~2] OR “freeze*”[tw] OR “freezing”[tw] OR “hypothermic preservation”[tiab:~2] OR “hypothermia*”[tw] OR “ice preservation”[tiab:~2] OR “refrigerat*”[tw] OR “low temperature storage”[tiab:~2] OR “cold ischemia*”[tw] OR “ischemia time*”[tw] OR “cold ischemia time”[tiab:~2] OR “cold ischemia times”[tiab:~2] OR “ex vivo preservation”[tiab:~2] OR “ex vivo preservations”[tiab:~2] OR “tissue preservation”[tiab:~2] OR “tissue preservations”[tiab:~2])

**Combined Search:**

(Set 1) AND (Set 2)

**Filter:**

Inception-2025

**Search Date:** 12/19/2025

**# of Results:** 58

**Embase Search Strategy**

**Set 1: Digit Replantation**

(‘replantation’/exp AND (‘finger’/exp OR ‘thumb’/exp)) OR (‘digit’ NEAR/2 ‘replant*’):ti,ab OR (‘finger’ NEAR/2 ‘replant*’):ti,ab OR (‘thumb’ NEAR/2 ‘replant*’):ti,ab OR (‘digit’ NEAR/2 ‘reattach*’):ti,ab OR (‘finger’ NEAR/2 ‘reattach*’):ti,ab OR (‘thumb’ NEAR/2 ‘reattach*’):ti,ab OR (‘digital’ NEAR/2 ‘replant*’):ti,ab OR (‘digital’ NEAR/2 ‘reattach*’):ti,ab OR (‘hand’ NEAR/2 ‘replant*’):ti,ab OR (‘hand’ NEAR/2 ‘reattach*’):ti,ab OR (‘digit amputat*’:ti,ab AND ‘replant*’:ti,ab)

**Set 2: Cryopreservation**

(‘cryopreservation’/exp OR ‘induced hypothermia’/exp OR ‘cryopreserv*’:ti,ab OR ‘cryo preserv*’:ti,ab OR (‘cold’ NEAR/2 ‘storage’):ti,ab OR (‘cold’ NEAR/2 ‘preservation’):ti,ab OR (‘frozen’ NEAR/2 ‘storage’):ti,ab OR ‘freeze*’:ti,ab OR ‘freezing’:ti,ab OR (‘hypothermic’ NEAR/2 ‘preservation’):ti,ab OR ‘hypothermia*’:ti,ab OR (‘ice’ NEAR/2 ‘preservation’):ti,ab OR ‘refrigerat*’:ti,ab OR (‘low’ NEAR/2 ‘temperature’ NEAR/2 ‘storage’):ti,ab OR (‘cold’ NEAR/2 ‘ischemia’):ti,ab OR (‘ischemia’ NEAR/2 ‘time’):ti,ab OR (‘ex’ NEAR/2 ‘vivo’ NEAR/2 ‘preservation’):ti,ab OR (‘tissue’ NEAR/2 ‘preservation’):ti,ab)

**Combined Search:**

(Set 1) AND (Set 2)

**Filter:**

Inception-2025

**Search Date:** 12/19/2025

**# of Results:** 61

**Web of Science Search Strategy**

**Set 1: Digit Replantation**

(TS=((digit NEAR/2 replant*) OR (finger NEAR/2 replant*) OR (thumb NEAR/2 replant*) OR (digit NEAR/2 reattach*) OR (finger NEAR/2 reattach*) OR (thumb NEAR/2 reattach*) OR (digital NEAR/2 replant*) OR (digital NEAR/2 reattach*) OR (hand NEAR/2 replant*) OR (hand NEAR/2 reattach*) OR ((digit NEAR/2 amputation) AND replant*)))

**Set 2: Cryopreservation**

(TS=(cryopreserv* OR cryo NEAR/2 preservation OR cold NEAR/2 storage OR cold NEAR/2 preservation OR frozen NEAR/2 storage OR freeze* OR freezing OR hypothermic NEAR/2 preservation OR hypothermia* OR ice NEAR/2 preservation OR refrigerat* OR low NEAR/2 temperature NEAR/2 storage OR cold NEAR/2 ischemia OR ischemia NEAR/2 time OR ex NEAR/2 vivo NEAR/2 preservation OR tissue NEAR/2 preservation))

**Combined Search:**

(Set 1) AND (Set 2)

**Filter:**

Inception-2025

**Search Date:** 12/19/2025

**# of Results:** 68

**Scopus Search Strategy**

**Set 1: Digit Replantation**

(TITLE-ABS-KEY((“digit” W/2 “replant*”) OR (“finger” W/2 “replant*”) OR (“thumb” W/2 “replant*”) OR (“digit” W/2 “reattach*”) OR (“finger” W/2 “reattach*”) OR (“thumb” W/2 “reattach*”) OR (“digital” W/2 “replant*”) OR (“digital” W/2 “reattach*”) OR (“hand” W/2 “replant*”) OR (“hand” W/2 “reattach*”) OR ((“digit” W/2 “amputation”) AND “replant*”)))

**Set 2: Cryopreservation**

(TITLE-ABS-KEY(“cryopreserv*” OR (“cryo” W/2 “preservation”) OR (“cold” W/2 “storage”) OR (“cold” W/2 “preservation”) OR (“frozen” W/2 “storage”) OR “freeze*” OR “freezing” OR (“hypothermic” W/2 “preservation”) OR “hypothermia*” OR (“ice” W/2 “preservation”) OR “refrigerat*” OR (“low” W/2 “temperature” W/2 “storage”) OR (“cold” W/2 “ischemia”) OR (“ischemia” W/2 “time”) OR (“ex” W/2 “vivo” W/2 “preservation”) OR (“tissue” W/2 “preservation”)))

**Combined Search:**

(Set 1) AND (Set 2)

**Filter:**

Inception-2025

**Search Date:** 12/19/2025

**# of Results:** 109
