# Supplementary material for: Current Evidence of Digital and Hand Replantation Following Cold Ischemia or Cryopreservation: A Systematic Review
Source: Microsurgery. 2026 Nov 11;46(8):e70323. doi: 10.1002/micr.70323 (PMC13754630; doi:10.1002/micr.70323)
Supplement: Supplementary file 3 [file MICR-46-e70323-s003.docx]

**Supplementary Material 2. Outcomes of Each Individual Study**

| First Author, Year | Study Design | Total Patients | Age | Gender |
| --- | --- | --- | --- | --- |
| Li 2008 | Retrospective Case Series | 211 | 26.2 | Male (n = 117); Female (n = 94) |
| Lin 2010 | Retrospective Case Series | 14 | 32 ± 10.7 | Male n = 14) |
| May 1986 | Retrospective Case Report | 1 | 21 | Male (n = 1) |
| VanderWilde 1992 | Retrospective Case Report | 1 | 15 | Female (n = 1) |
| Wang 2019 | Retrospective Case Series | 2 | 23 ± 5.7 | Male (n = 2) |
| Wang 2020 | Retrospective Case Series | 2 | 42.5 ± 3.5 | Male (n = 2) |
| Watson 2018 | Retrospective Case Report | 1 | 38 | Male (n = 1) |
| Wei 1988 | Retrospective Case Report | 1 | 24 | Female (n = 1) |
| Woo 2015 | Retrospective Case Series | 20 | 45 ± 7.0 | Male (n = 17); Female (n = 3) |
| Zhou 1991 | Retrospective Case Series | 2 | 38.5 ± 7.8 | Male (n = 1); Female (n = 1) |

Age was listed as individual age for case reports or mean (± standard deviation, if available) for case series.

| First Author, Year | Mechanism of Injury | Digit Replants (n = 279) | Hand Replants (n = 8) | Follow-up |
| --- | --- | --- | --- | --- |
| Li 2008 | Crush or avulsion (n = 92), clean cut (n = 65), blunt cut (n = 54) | 211 | 0 | 4 weeks |
| Lin 2010 | Guillotine or localized crush (n = 14) | 23 | 2 | 2 years |
| May 1986 | Metal press crush avulsion (n = 1) | 8 | 0 | 35 months |
| VanderWilde 1992 | Sharp blade injury (n = 1) | 0 | 1 | 30 months |
| Wang 2019 | Self-inflicted sharp amputation (n = 2) | 2 | 0 | 14.5 years |
| Wang 2020 | Bite injury (n = 1); amputation injury (n = 1) | 2 | 0 | 1 year |
| Watson 2018 | Guillotine w/ machete (n = 1) | 0 | 1 | 30 months |
| Wei 1988 | Sharp amputation with paper-cutting machine (n = 1) | 3 | 0 | 8 months |
| Woo 2015 | Guillotine (n = 8), crush (n = 7), avulsion (n = 5) | 28 | 4 | 26 months |
| Zhou 1991 | Severed by cotton gin (n = 1); tractor injury (n = 1) | 2 | 0 | 3.5 years |

Follow-up was listed as individual for case reports or mean for case series.

| First Author, Year | Causes of Replantation Failure (n = 52 digit, 1 hand) | Overall Complications of Successful Replantation (n = 8 digit, 0 hand) |
| --- | --- | --- |
| Li 2008 | Digits: Tissue necrosis (n = 39) | None |
| Lin 2010 | Digits: NR (n = 8); Hands: Venous congestion (n = 1) | None |
| May 1986 | Ischemia (n = 1) | Digits: Thrombosis requiring reoperation (n = 5)* |
| VanderWilde 1992 | None | None |
| Wang 2019 | None | None |
| Wang 2020 | None | None |
| Watson 2018 | None | None |
| Wei 1988 | None | Digits: Edema (n = 3) |
| Woo 2015 | Digits: NR (n = 4) | None |
| Zhou 1991 | None | None |

*6 cases of thrombosis were reported but it could not be determined if the digit that experienced ischemia and subsequent failure also experienced thrombosis so only 5 cases of thrombosis are calculated in the overall complications of successful replantation.

NR: not reported.
